# The p21-activated kinases in neural cytoskeletal remodeling and related neurological disorders

**DOI:** 10.1007/s13238-020-00812-9

**Published:** 2020-12-11

**Authors:** Kaifan Zhang, Yan Wang, Tianda Fan, Cheng Zeng, Zhong Sheng Sun

**Affiliations:** 1 Beijing Institutes of Life Science, Chinese Academy of Sciences, Beijing, 100101 China; 2 Institute of Genomic Medicine, Wenzhou Medical University, Wenzhou, 325000 China; 3 CAS Center for Excellence in Biotic Interactions, University of Chinese Academy of Sciences, Beijing, 100049 China; 4 State Key Laboratory of Integrated Management of Pest Insects and Rodents, Chinese Academy of Sciences, Beijing, 100101 China

**Keywords:** p21-activated kinases, expression pattern, synaptic cytoskeletal remodeling, neuronal function, neurological diseases

## Abstract

The serine/threonine p21-activated kinases (PAKs), as main effectors of the Rho GTPases Cdc42 and Rac, represent a group of important molecular switches linking the complex cytoskeletal networks to broad neural activity. PAKs show wide expression in the brain, but they differ in specific cell types, brain regions, and developmental stages. PAKs play an essential and differential role in controlling neural cytoskeletal remodeling and are related to the development and fate of neurons as well as the structural and functional plasticity of dendritic spines. PAK-mediated actin signaling and interacting functional networks represent a common pathway frequently affected in multiple neurodevelopmental and neurodegenerative disorders. Considering specific small-molecule agonists and inhibitors for PAKs have been developed in cancer treatment, comprehensive knowledge about the role of PAKs in neural cytoskeletal remodeling will promote our understanding of the complex mechanisms underlying neurological diseases, which may also represent potential therapeutic targets of these diseases.

## Introduction

Most neurons receive information from other neurons via synapses mainly formed on the surface of dendritic spines. Thus, the morphological plasticity and density of dendritic spines are crucial for the physiological functions of neurons. Dendritic spines consist of a network of actin filaments, whose polymerization and depolymerization can govern spine function and regulate synaptic plasticity. Dysfunction of synaptic cytoskeletal remodeling is closely related to diverse brain disorders (Yan et al., 2016). As the initially identified and main downstream effectors of the Rho family small GTPases Cdc42 and Rac1, PAKs (p21-activated kinases) represent a family of serine/threonine kinases that can connect cytoskeletal dynamics, mechanical forces, and neuron morphology (Daniels and Bokoch, 1999; Nobes and Hall, 1999). It is widely recognized that PAKs play a potent and diversified role in controlling the morphology, motility, and fate of neurons to maintain the normal function of dendritic spines (Manser et al., 1994; Jaffer and Chernoff, 2002; Rane and Minden, 2014).

**Figure 1:**
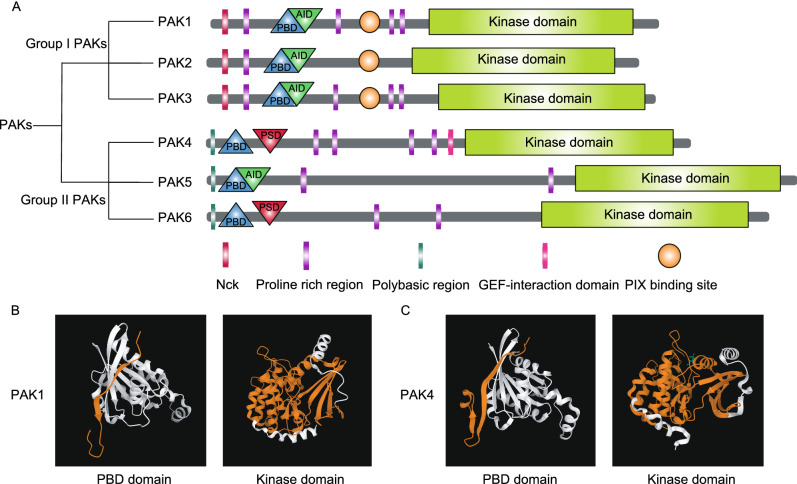
**Domains and structural features of PAKs**. (A) Schematic representation of domains of PAKs; PAKs are classified into two group, group I PAKs and group II PAKs. All members of PAKs contain a conserved N-terminal p21-binding domain (PBD) and a C-terminal kinase domain (KD). (B) Protein structure of PBD and KD in PAK1. PBD binds Cdc42/Rac1. (C) Protein structure of PBD and KD in PAK4

To date, six PAKs have been identified in mammals. Based on their structural differences and sequence homologies, PAKs are classified into two groups. PAK1, PAK2, and PAK3 belong to group I, whereas PAK4, PAK5, and PAK6 belong to group II (Fig. 1) (Sells and Chernoff, 1997; Knaus and Bokoch, 1998; Daniels and Bokoch, 1999). Overall, group I PAKs have higher sequence similarity than group II PAKs in the PBD (p21-binding domain) and kinase domains, but differ throughout their other domains (Eswaran et al., 2008). Specifically, group I PAKs contain one PBD that overlaps an AID (autoinhibitory domain) in the N-terminus, and one serine/threonine kinase domain in the C-terminus (Knaus and Bokoch, 1998). The PBD serves as the binding site of active Cdc42 and Rac GTPases (Jaffer and Chernoff, 2002). The AID binds to the kinase domain of another PAK to form an inactive homodimer (Rane and Minden, 2014). The homodimer is dissociated once Rho GTPases bind to the PBD domain, which then leads to conformational reorganization and subsequent autophosphorylation at multiple sites (Rane and Minden, 2014). The autophosphorylation in the kinase domain allows the binding of group I PAKs to the substrate and exerting of its catalytic function in a monomeric conformation (Jaffer and Chernoff, 2002). There are other domains in the N-terminal of group I PAKs, including conserved SH3 (Src homology 3)-binding motifs for binding to adaptor proteins, such as NCK1 (NCK adaptor protein 1) and GRB2 (growth factor receptor-bound protein 2), and the PIX binding domain for PAK-interacting exchange factor/Cool (PIX/Cool), such as nucleotide-exchange factor PIX (Lei et al., 2000; Parrini et al., 2002; Pirruccello et al., 2006). In contrast, group II PAKs contain a PBD and an AID (for PAK5) or a PSD (pseudosubstrate domain) (for PAK4 and PAK6) in the N-terminus, and a serine/threonine kinase domain in the C-terminus (Ha et al., 2012; Tabanifar et al., 2016). Group II PAKs are monomers in the inactive state, and the PSD or AID mediates this inactive state by preventing their substrates from entering the catalytic site (Gao et al., 2013; Wang et al., 2013). Group II PAKs show greater binding affinity to Cdc42 than to Rac1 (Arias-Romero and Chernoff, 2008). Although the PBD is present in group II PAKs, whether its role is similar to that in group I PAKs remains debatable. Normally, the contact between Cdc42 and the PBD of group II PAKs alters their intracellular location (Chenette et al., 2006). In addition to the PBD, additional interactions of Cdc42-PAK may suppress the kinase activity and contribute to the regulation of its inactive state (Fig. 1). For example, Cdc42 binds to the PBR (polybasic region) and C-terminal lobe of PAK4 and inhibits the kinase activity *in vitro* (Ha and Boggon, 2018).

PAKs serve as key regulators of cell growth, cytoskeletal dynamics, cell morphology, cell migration, and cell cycle progression, as well as of death and survival events. Previous studies have indicated that dysfunction of PAKs can result in cancer development and progression, and small molecules that regulate PAK activity have been used for the treatment of multiple cancers (Radu et al., 2014). Recently, emerging evidence indicates that PAKs are essential for spine morphogenesis, neural plasticity, and brain-related functions and behaviors. In particular, the actin signaling networks mediated by PAKs represent a common pathway frequently affected in multiple neurodevelopmental and neurodegenerative disorders, including autism, mental retardation, and schizophrenia (Allen et al., 1998; Zhao et al., 2006; Huang et al., 2011; Dolan et al., 2013; Hayashi-Takagi et al., 2014; Harms et al., 2018; Wang et al., 2018; Horn et al., 2019). In this review, we summarize the recent findings about the functions of PAKs in the nervous system, including the structures and expression patterns of PAKs during brain development. We then discuss the specific functions of PAKs in neural development, migration, polarity, differentiation, and cell fate determination, as well as synaptic and neural cytoskeleton-related synaptic plasticity. Neurological disorders associated with PAK dysfunction are also discussed by integrating multiple levels of evidence from mouse models and human genetics. A comprehensive review of the function of PAKs in synaptic cytoskeleton-related neural function will promote our understanding of the complex molecular mechanisms underlying neurological diseases and help us determine the series of events that lead to the progression of these diseases.

## Expression of PAKs In the Nervous System During Brain Development

**Figure 2:**
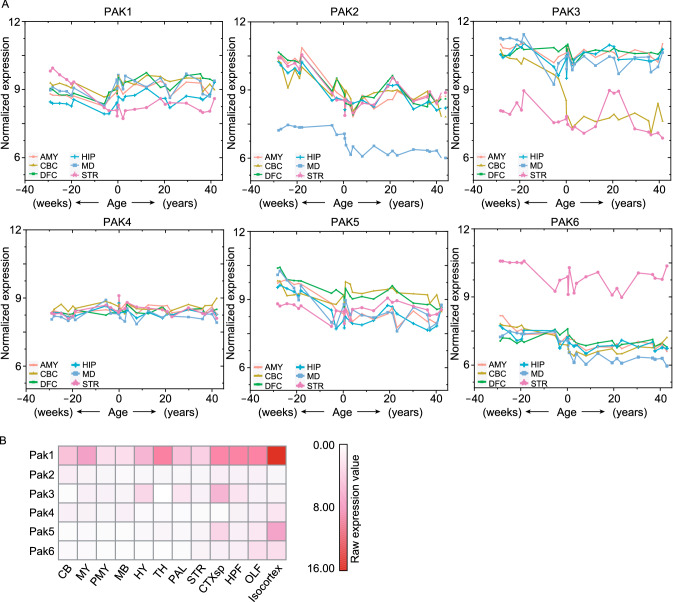
***PAKs*** **show a differential expressional pattern from the fetal period to after birth in the human and mouse brain**. We downloaded transcriptome expression data profiling in the HBT database, with the transcriptome data read by R package “oligo”. (A) Scatter plot representation of *PAKs* expression using transcriptome data. DFC, dorsolateral prefrontal cortex; HIP, hippocampus; AMY, amygdaloid complex; MD, mediodorsal nucleus of thalamus; CBC, cerebellar cortex; STR, striatum. (B) Heatmap presents the expression of each *Pak* in adult mouse brain in the sagittal plane. OLF, olfactory areas; HPF, hippocampal formation; CTXsp, cortical subplate; STR, striatum; PAL, pallidum; TH, thalamus; HY, hypothalamus; MB, midbrain; P, pons; MY, medulla; CB, cerebellum

Considering little is known about the expression of PAKs at different developmental stages in the nervous system, we characterized the expression patterns of *PAK* genes in the human brain by analyzing data from the HBT (Human Brain Transcriptome) database (Johnson et al., 2009; Kang et al., 2011; Pletikos et al., 2014) from the fetal period to after birth. We downloaded transcriptome expression data with the probe IDs corresponding to the *PAK* gene profiling by array on the Affymetrix Human Exon 1.0 ST Array [transcript (gene) version] Platform in the HBT database from 6 brain regions of 59 donors. The expression value of each *PAK* gene was averaged using data from both the left and right brain hemispheres of same-age donors. We found that most *PAK* levels showed a decrease at the late prenatal stage, but each *PAK* presented a differential pattern in different brain regions, including the DFC (dorsolateral prefrontal cortex), hippocampus, amygdala, mediodorsal nucleus of the thalamus, cerebellar cortex, and striatum, from 40 weeks before birth to 40 years after birth (Fig. 2A). Specifically, *PAK1* was highly expressed at every stage of life, especially after birth until adulthood, and it showed a slightly decreasing trend in the striatum, but a generally increasing trend in other regions of the brain during development. In contrast, *PAK2* showed high expression levels during the fetal period that were gradually decreased and maintained at a stable low level after birth. *PAK3* showed low expression in the striatum and high expression in other brain regions during the fetal period but an obvious decrease in the cerebellar cortex during development. In general, *PAK4* expression was relatively stable during the fetal period and after birth. The expression trend of *PAK5* was similar to that of *PAK2*, with the exception that its decline range was slightly lower than that of *PAK2*. In contrast to *PAK3*, *PAK6* showed relatively high expression in the striatum and low expression in other brain regions at every stage of life (Fig. 2A). We also analyzed the expression patterns of *Pak* genes in the adult mouse brain by analyzing the data from *in situ* hybridization dataset in the Mouse Brain module from the Allen Brain Atlas database (Lein et al., 2007) and found that the expression pattern of *Paks* at the adult stage in the mouse brain was generally similar to the pattern in the human brain. For example, *Pak1* was highly expressed in all detected brain regions; however, the *Pak2* level was relatively low in the adult mouse brain (Fig. 2B).

**Figure 3:**
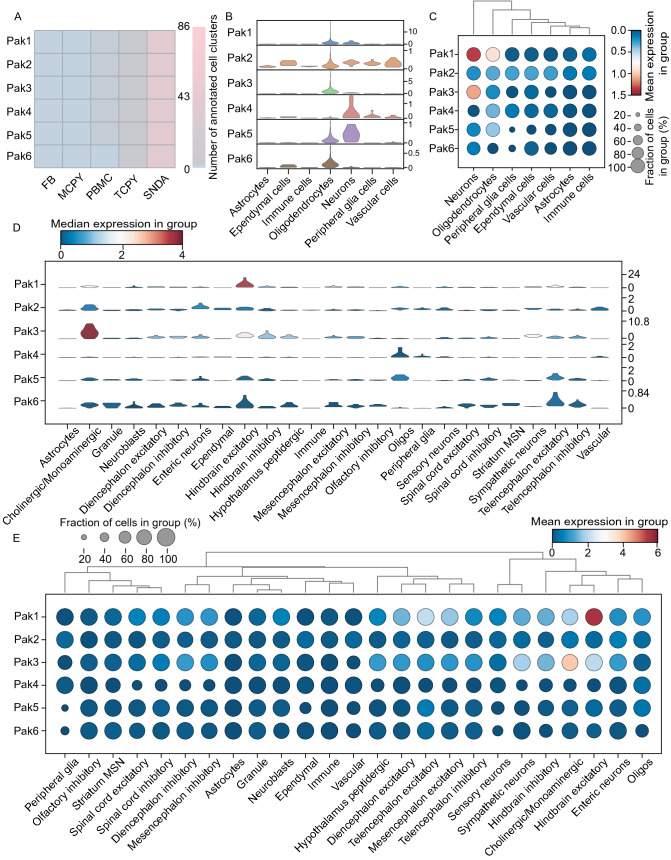
**The expression of PAKs in specific cell type and brain region**. (A) Heatmap shows the cell number of each PAK in five specific cell types in the human brain using the R package “pheatmap”. FB, fibroblast; MCPY, motor cortex pyramidal neurons; PBMC, peripheral blood mononuclear cells; TCPY, temporal cortex pyramidal neurons; SNDA, substantia nigra dopamine neurons. (B) Violin plotting shows the expression level of each *Pak* in seven cell types in the mouse brain. The Y-axis represents the expression level of cell type. (C) Dot plotting shows the mean expression and fraction levels of each *Pak* in seven cell types in the mouse brain. (D)Violin plotting shows the expression level of each *Pak* in 20 specific cell types in the mouse brain using the Python package ‘scanpy’ versions 1.6.0 and 1.5.1. MSN, medium spiny neurons. (E) Dot plotting shows the mean expression and fraction levels of each *Pak* in 20 specific cell types in the mouse brain. MSN, medium spiny neurons

To analyze the cell-type-specific expressions of *PAKs* in the human brain, we downloaded RNA-Seq data of 99 human postmortem brains and seven cell-type samples from the BRAINcode database (Dong et al., 2018). We found that *PAKs* were highly expressed in the clusters of SNDA (substantia nigra dopamine neurons) and TCPY (temporal cortex pyramidal neurons), but relatively low expression was found in the clusters of PBMC (peripheral blood mononuclear cells), MCPY (motor cortex pyramidal neurons), and FB (fibroblast) (Fig. 3A). To analyze the cell-type-specific expressions of *Paks* in the mouse brain, the single cell RNA-seq data from Mousebrain.org, including 133 mouse samples from 19 regions in the central nervous system and peripheral nervous system, were analyzed by droplet microfluidics (10× Genomics Chromium) (Zeisel et al., 2018). We first calculated *Paks* expression in seven cell types, including astrocytes, ependymal cells, immune cells, neurons, oligodendrocytes, peripheral glial cells, and vascular cells (Fig. 3B and 3C). While *Pak1* and *Pak3* were highly enriched in the cluster of neurons, *Pak2* expression showed relatively higher levels in ependymal cells, neurons, and vascular cells than in other cell types. Notably, *Pak1*, *Pak4* and *Pak5* also exhibited relatively high expression in oligodendrocytes (Fig. 3B and 3C), suggesting their functions in non-neuronal cells. To provide more specific cell-type information, *Pak* expression in 20 cell types was also analyzed and calculated using the same data (Fig. 3D and 3E). We found that *Paks* were expressed in various neuronal and non-neuronal cells, including cholinergic and monoaminergic neurons, hindbrain excitatory neurons, oligodendrocytes and telencephalon excitatory neurons. Specifically, *Pak1* was relatively enriched in hindbrain excitatory neurons, *Pak2* in the clusters of cholinergic and monoaminergic neurons and enteric neurons, *Pak3* in cholinergic and monoaminergic neurons, *Pak4* in oligodendrocyte clusters, *Pak5* in the clusters of oligodendrocytes and telencephalon excitatory neurons, and *Pak6* in the clusters of hindbrain excitatory neurons and telencephalon excitatory neurons. These findings suggest that different PAKs may exert different functions in the brain.

## Function of PAKs in Neural Cytoskeletal Remodeling

The F-actin (filamentous actin) cytoskeleton is the main driving force behind dendritic spine remodeling and maintenance of synaptic plasticity. Cofilin is a major actin-depolymerizing factor that regulates the actin cytoskeleton dynamics and phosphorylation of serine residue at 3 (Ser 3) can inhibit its actin-depolymerizating activity (Yang et al., 1998). PAK can phosphorylate cofilin at Ser3 via LIMK1/2 (LIM motif-containing protein kinases 1 and 2) to prevent F-actin depolymerization (Arber et al., 1998; Yang et al., 1998; Li et al., 2013; Wang et al., 2018). Due to their high expression in the adult brain, the group I PAKs, particularly PAK1 and PAK3, are the most extensively studied mammalian PAKs for their function in brain and behavior. However, emerging studies indicate that other PAKs also play important roles in the formation and maintenance of dendritic spines and activity-dependent synaptic plasticity via regulating actin cytoskeleton dynamics (Manser et al., 1994; Rane and Minden, 2014). To date, PAKs have been found to be involved in diverse neural developmental processes, such as PAK1/2/3/6 in neuronal migration (Causeret et al., 2009; Pensold et al., 2017; Wang et al., 2018; Liu et al., 2019), PAK1/3/6 in neurite outgrowth (Cobos et al., 2007; Li et al., 2013; Civiero et al., 2015), PAK1 in neuronal polarity (de la Torre-Ubieta et al., 2010), PAK1/3 in neuronal differentiation (Li et al., 2013; Maglorius Renkilaraj et al., 2017), and PAK1/4 in axonal development (Hing et al., 1999; Qu et al., 2003; Chen et al., 2009). In the following sections, we summarize how PAKs are involved in maintaining the structural and functional plasticity of dendritic spines by regulating actin cytoskeletal networks. A detailed summary of the function of each PAK in neural development and processes is summarized in Table 1. Established mouse models for each member of the PAK family are also summarized in Table 2.

**Table 1:** Functions of PAKs in diverse neural development processes

Functions	Species	PAK1	PAK2	PAK3	PAK4	PAK5	PAK6
Neuronal migration	Mouse	Cortical neurons (Zhong et al., 2003)	Cortical neuron (Wang et al., 2018)	Cortical neurons;GABAergic Ins (Liu et al., 2019; Cobos et al., 2007)	Spinal cord MNs; ventral Ins (Qu et al., 2003)	–	GABAergic interneurons (Pensold et al., 2017)
	*C*. *elegans*	Hermaphrodite-specific neurons (Lin et al., 2001)	–	–	–	–	–
	Xenopus	–	–	Neural plate neurons (Souopgui et al., 2002)	–	–	–
Neurite outgrowth	Mouse	Cortical neurons (Li et al., 2013)	–	MGE-derived Ins (Cobos et al., 2007)	–	–	POA-derived cells; cortical neurons (Pensold et al., 2017)
Neuronal polarity and positioning	Rat	Primary granule neurons (de la Torre-Ubieta et al., 2010)	–	–	–	–	–
	Drosophila	MNs (Kamiyama et al., 2015)	–	–	–	–	–
Axonal development	Mouse	Hippocampal neurons (Li et al., 2013)	–	–	Neural progenitors (Qu et al., 2003)	–	–
	C. elegans	MNs (Lucanic et al., 2006)	–	–	–	–	–
	Drosophila	Photoreceptor cells (Hing et al., 1999)	–	–	–	–	–
Neuronal differentiation/Cell Fate determination	Mouse	Cortical neurons (Li et al., 2013)	–	GABAergic INs; OD precursors (Cobos et al., 2007; Maglorius Renkilaraj et al., 2017)	Spinal cord MNs; ventral Ins (Qu et al., 2003)	–	–
	Xenopus	–	–	Neural progenitors (Souopgui et al., 2002)	–	–	–

Notes: –, not determined; INs, interneurons; OD, oligodendrocyte; MNs, motor neurons

**Table 2:** *PAK* Mutations identified in patients with diverse neurological diseases

	Chr.	Position	Alleles	Origin	Consequence	Diseases/Phenotypes	References
PAK1	Chr11	77336213	T>C	*De novo*	Missense	ID with macrocephaly, seizures, speech delay	Harms et al., 2018; Horn et al., 2020
		77379288	T>C				
		77474957	G>A / G>T	–	Intron variant	SCZ	Jiang et al., 2017
		77322663	A>C / A>G		3′ UTR variant		
**PAK2**	Chr3	q29	Deletion	*De novo/*inherited	–	3q29 microdeletion syndrome	Willatt et al., 2005
		196554151	C>T	*De novo*	Nonsense	Autism	Wang et al., 2018
		196555271	C>T	Maternal inherited	Missense		
		196555271	G>A	Paternal inherited			
**PAK3**	ChrX	111142119	C>T	–	Missense	X-Linked MR, ID	Bienvenu et al., 2000
		111194402	C>A	Paternal inherited			Gedeon et al., 2003
		111196488	C>T	–	Nonsense		Allen et al., 1998
		111196570	G>C	Inherited	Missense		Peippo et al., 2007
		111142200	A>G		Splice region variant		Rejeb et al., 2008

Notes: –, not determined; MR, mental retardation; SCZ, schizophrenia; ID, intellectual disability; Chr, chromosome

### Synaptic plasticity

Synaptic plasticity is closely related to the morphology and density of dendritic spines, wherein F-actin is highly expressed and forms a complex cytoskeletal network. PAKs can control the polymerization and depolymerization of F-actin and play an indispensable role in synaptic plasticity (Kreis and Barnier, 2009). Abnormal activation of Rac-PAK or Rac-GEF/PAK signaling can result in aberrant actin polymerization, increased spine density, and the suppression of spine maturation (Nobes and Hall, 1995; Nobes and Hall, 1999; Byrne et al., 2016). Mice overexpressing an autoinhibitory-negative PAK in the postnatal forebrain showed fewer spines, and synapse distribution in these mice exhibited a shift toward synapses of larger size, suggesting the direct contribution of PAK catalytic activity to synaptic plasticity (Hayashi et al., 2004). The Rac/PAK signaling pathway is also required for the rapid stabilization of newly formed polymers of actin filaments induced by stimulation, and thus for activity-induced changes of spine morphology and synaptic plasticity (Chen et al., 2010). In addition to its regulation of actin dynamics at postsynaptic spines, PAK activation also regulate presynaptic actin network and is required for the formation of actin-based filopodia in presynaptic terminals related to long-term memory (Udo et al., 2005).

PAKs dysfunction has induced abnormal synaptic morphology and functional impairments in synaptic plasticity and behavior in mice. For example, *Pak1* knockout mice showed deficits in spine actin filaments and NMDA-induced cofilin activity as well as dramatically reduced hippocampal LTP (long-term potentiation) (Asrar et al., 2009). Mice lacking the gene *Pak3* exhibited reduction in phosphorylated CREB levels but normal actin cytoskeleton, possibly due to the compensatory roles of other PAK family members in the brain (Meng et al., 2005). Consequently, a selective deficit in hippocampal late-phase LTP as well as deficiencies in hippocampus-independent learning tasks were found in the *Pak3* knockout mice (Meng et al., 2005). PAK3 can be specifically recruited to the head of the activated spine by activity to control activity-mediated local spine growth and synaptic connectivity. Inhibition of PAK3 in a rat hippocampal slice induced the growth of new, unstable spines and an impairment of activity-dependent spine stabilization (Dubos et al., 2012). Notably, *Pak1*/*Pak3* double-knockout mice showed a robust microcephaly phenotype in postnatal brain growth (Huang et al., 2011). Increased neural and glial cell density but reduced dendritic arbors and axons and enlarged individual synapses in the CA1 region of the hippocampus, along with enhanced basal synaptic transmission and reduced LTP and LTD (long-term depression), were also evident in the double-knockout mice (Huang et al., 2011). Moreover, our recent study indicated that PAK2 dysfunction resulted in decreased synapse densities, attenuated LTP, and autism-related behaviors in mice (Wang et al., 2018).

In addition to neuronal morphology, PAKs regulate synaptic transmission and trafficking. For example, PAK3 controls the surface trafficking of the major excitatory receptor GluA1 AMPAR (AMPA receptor) subunit in neurons (Hussain et al., 2015). PAKs can interfere with Shank3 to regulate NMDAR membrane delivery or stability (Duffney et al., 2013). PAKs can also control synaptic GABA(A)R surface stability via GIT1 (G protein-coupled receptor kinase interacting ArfGAP 1)/βPIX signaling (Smith et al., 2014). PAK signals function as the downstream of the scaffolding protein SAP102 and EphB2 to regulate synaptic AMPAR trafficking and localization in the neonatal cortex of mice (Murata and Constantine-Paton, 2013). Moreover, PAKs can function downstream of AMPK (AMP-activated protein kinase) and regulate the activity of excitatory synapses in AgRP (agouti-related peptide) neurons to produce fasting-induced plasticity (Banko et al., 2011; Kong et al., 2016). When AgRP neurons are activated, AMPK can phosphorylate PAK2 and its target LIMK2. Inhibition of PAK by overexpressing AID in the arcuate nucleus greatly decreased AgRP neuron activity, mEPSCs (miniature excitatory postsynaptic currents) frequency and body weight of fasted mice, suggesting that PAKs is required for fasting- and AMPK-induced effects on excitatory synaptic plasticity (Kong et al., 2016). PAK1 can maintain the balance of excitation and inhibition through endocannabinoid signaling (Xia et al., 2016). PAK1 up-regulates synaptosomal COX-2 (cyclooxygenase-2) expression, which then decrease endocannabinoid signaling and facilitate inhibitory synaptic transmission by increasing GABA transmission. PAK1 disruption in mice resulted in suppressed inhibitory neurotransmission through reducing COX-2 expression and increasing endocannabinoid secretion (Xia et al., 2016). PAK-cofilin-mediated actin PAK signaling regulates synaptic function in the entorhinal cortical to dentate gyrus circuit (EC-DG) and play a critical role in social recognition memory retrieval (Leung et al., 2018). Inducible disruption of PAK signaling impairs synaptic transmission at the EC-DG terminals and selectively impairs social recognition memory (Leung et al., 2018). Moreover, PAK5 can phosphorylate Pacsin1 and Synaptojanin1, two proteins that regulate synaptic vesicle endocytosis and recycling, suggesting the role of PAK5 in synaptic vesicle trafficking (Strochlic et al., 2012).

**Figure 4:**
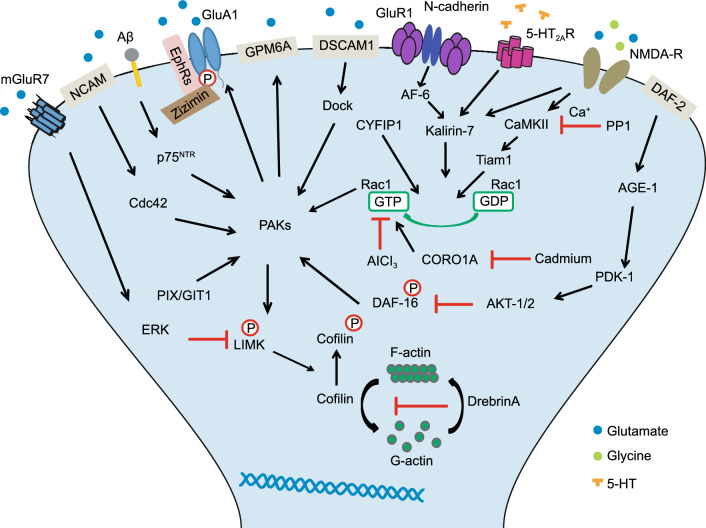
**Graphic models of PAK signaling pathways in synaptic plasticity**. The upstream activators of PAKs include Cdc42, Rac1, Dock, p75NTR, DAF-16 and PIX/GIT1 and the downstream factors contain GPM6A, GluA1 and LIMK (Henderson and Johnson, 2001; Kennedy et al., 2013; Li et al., 2013; Murata and Constantine-Paton, 2013; Gu et al., 2014; Hussain et al., 2015; Kamiyama et al., 2015; Ramos et al., 2015; Byrne et al., 2016; Kim et al., 2017; Santini et al., 2017; Leung et al., 2018; Feng et al., 2019). The main effect of PAKs is to regulate the formation of the cytoskeleton by activation of the LIMK-Cofilin pathway. The disruption of PAKs-related pathways is closely associated with diverse neurological disorders

PAKs can regulate synaptic plasticity by affecting important signaling pathways. For example, the scaffolding proteins MAGUKs (membrane-associated guanylate kinases), such as SAP102, can interact with EphB2 and Kalirin-7, a neuronal exchange factor for small GTPase, to activate their key downstream PAK signals (Murata and Constantine-Paton, 2013). The EphB/SAP102/PAK signaling can regulate synaptic AMPAR trafficking and localization in the neonatal cortex of mice and thus has critical roles in cortical synapse development (Murata and Constantine-Paton, 2013). The FMRP-CYFIP1-eIF4E inhibitory complex can maintain the appropriate polymerization and stabilization of actin filaments in response to synaptic activity by inhibiting Rac1-PAK1/2 activation, which is fundamental for generating multiple forms of long-term synaptic plasticity at glutamatergic synapses (Santini et al., 2017). The GIT1/βPIX/Rac1/PAK pathway can modulate F-actin and plays a crucial role in maintaining surface GABA(A)R levels and thus inhibits synaptic plasticity in the brain (Smith et al., 2014). PAK1 also plays key roles in synaptogenesis and spine morphogenesis by participating in ephrinB-EphB receptor signaling (Penzes et al., 2003). PAKs can also form a complex with pre/postsynaptic proteins to regulate synaptic plasticity. For example, PAKs can form a complex with Scribble, β-PIX, and GIT1, to regulate dendritic spine development via MLC (myosin II regulatory light chain). This complex can bind to NOS1AP, leading to Rac activation and altered spine morphology (Richier et al., 2010). Similarly, in *Drosophila*, Neto (neuropilin and tolloid-like)-β can regulate the accumulation of PSD-associated PAKs by interacting with a large protein complex containing dPIX and Dock, which are crucial to the composition of neural circuits and the long-term plasticity of learning and memory at the neuromuscular junction (Ramos et al., 2015). However, most of the mechanisms underlying the relationship between PAKs and pre/postsynaptic proteins remain unclear. Figure 4 shows the detailed signaling pathways mediated by the PAK-related cytoskeleton in dendritic spines.

### Neuronal migration

Neuronal migration from the birthplace to final location occurs in newly formed neurons and is critical for nervous system development (Guo et al., 2007; Marin et al., 2010). During the key stages of cortical development, group I PAKs are activated and expressed in migrating neurons, thereby regulating neuronal migration. For example, PAK1 controls the correct orientation and morphology as well as radial migration of neurons in the developing cerebral cortex (Zhong et al., 2003). PAK1 also promotes the formation of polarized lamellipodia in migrating neurons and is essential for maintaining correct neocortical laminar organization (Causeret et al., 2009). Inhibition of PAK1 changes the morphology of migrating neurons, causing them to accumulate in the intermediate zone and deep layers of the cortex of mice (Causeret et al., 2009). The binding of the Ras prenylation sequence (CAAX-box) to the C-terminus of PAK1 is essential for its membrane activity (Jacobs et al., 2007). Overexpression of PAK1-CAAX caused neurons to reside in the intermediate zone of mice (Causeret et al., 2009). Notably, we found that PAK2 regulated the migration of neurons to the cortical plate in the developing cortex. PAK2 deficiency resulted in a dampened actin network and fewer neurons to migrate into the cortical plate (Wang et al., 2018).

RGCs (radial glial cells) are the principal subtype of neuronal progenitors and can produce most cortical neurons in the neocortex (Gotz and Barde, 2005). Zeb1 is expressed in RGCs during neocortical neurogenesis. It can bind to PAK3, which is relatively enriched in RGCs (Fig. 2B), and the methyltransferase PRMT5 to form a repressing complex that regulates radial migration and neuronal multipolar-bipolar transition in the developing mouse cortex (Liu et al., 2019). PAK3 is dramatically upregulated in *Zeb1* knockout mice. Overexpression of PAK3 in wild-type cortical neurons showed the same phenotype as those in *Zeb1* knockout mice, whereas *Pak3* knockout rescued the abnormal migration of cortical neurons in *Zeb1* knockout mice (Liu et al., 2019). Moreover, PAK3 expression is absent in MGE (medial ganglionic eminence)-derived migratory interneurons, but is upregulated as neurons differentiate (Cobos et al., 2007). PAK3 overexpression in the MGE can inhibit the tangential migration of interneurons to the neocortex (Cobos et al., 2007). In humans, a recent study highlighted that the function of PAK3 in cell migration was associated with brain morphological changes in patients with intellectual disability and corpus callosum agenesis (Duarte et al., 2020).

Migration of GABAergic interneuron from their origin sites in the subpallium of the embryonic POA (preoptic area) to distant targets, such as the cerebral cortex, is a critical step in GABAergic interneuron development (Martini et al., 2009; Cooper, 2013). DNA methylation by DNMTs (DNA methyltransferases) is increased during the migration of active immature GABAergic interneurons (Pensold et al., 2017). DNMT1-positive cells showed significantly reduced levels of PAK6 expression, but cells expressing *Pak6* were presented in later stages of maturation of postmigratory POA-fated neurons. Suppression of PAK6 by DNMT1 in POA-derived migrating GABAergic cells can prevent premature neuritogenesis and preserve their migratory morphology (Pensold et al., 2017). The results suggest that PAK6 is involved in the migration of GABAergic interneurons.

In addition to mammalian systems, PAKs regulate neural migrations in other species. For example, PAK3 can promote neuronal differentiation and migration in *Xenopus laevis* (Souopgui et al., 2002). PAK1 regulates the migration of HSNs (hermaphrodite-specific neurons) in *Caenorhabditis elegans*. The transcription factor DAF-16/FOXO, a known target of IGF-1 (insulin/insulin-like growth factor-1) signaling, regulates the posterior-to-anterior migration of HSNs in developing embryos (Henderson and Johnson, 2001; Lee et al., 2001; Lin et al., 2001). PAK1 is a downstream regulator of insulin/IGF-1-DAF-16 signaling during HSN migration (Kennedy et al., 2013). Considering PAK1 can help guide the migration of HSNs by remodeling the actin cytoskeleton and/or regulating cell adhesion at hypodermal cell boundaries, the roles of PAK1 and FOXO in neuronal migration in higher organisms deserve further investigation (Kennedy et al., 2013). Whether other PAKs family members also function as the downstream of FOXO in neural migration remain to be investigated.

### Neurite outgrowth

The movement and dynamics of growth cones, which guide the rate and direction of neurite outgrowth, are largely dependent on the assembly and reorganization of filopodia and lamellipodia (Brown et al., 2000). Rho GTPases, such as Rac1 and Cdc42, mediate the formation of lamellipodia and filopodia. As the main downstream effectors of Rac1 and Cdc42, PAKs play an important regulatory role in neurite growth in diverse species.

In the growth cones of neurons, PAK1 can interact with the NCAM (neural cell adhesion molecule), a member of the Ig superfamily, to induce the activation of the PAK1-LIMK1-cofilin pathway and promote cytoskeletal remodeling processes and filopodium mobility (Li et al., 2013). NCAM activation results in the dephosphorylation of PAK1 at Thr212 and hyperphosphorylation of PAK1 at Ser199/204 and Thr423, which are beneficial to the promotion of PAK1 activity and the formation of the PAK1/PIX/Cdc42 complex (Nikolic et al., 1998; Rashid et al., 2001; Parrini et al., 2009). After autophosphorylation, activated PAK1 phosphorylates LIMK at Thr508, which in turn phosphorylates cofilin at Ser3, followed by a reduction in its actin-depolymerizing activity (Li et al., 2013). Increased actin polymerization promotes the movement of the growth cone and the generation of traction forces needed for neurite outgrowth (O’Donnell et al., 2009).

PAK3 contributes to the neurite outgrowth of cortical GABAergic and MGE-derived interneurons. *Pak3* knockout inhibits neurite growth in cortical GABAergic interneurons during their migration (Cobos et al., 2007). Downregulation of PAK3 rescues the inordinate growth of neurites in postmigratory MGE-derived interneurons in *Dlx1*/*2* mutants (Cobos et al., 2007). PAK6 plays an essential role in cytoskeletal organization, thereby affecting cell shape, motility, and adhesion (Civiero et al., 2015). PAK6 promotes neurite outgrowth in postmitotic POA cells, which are characterized by a significantly high expression of PAK6. PAK6 also promotes neurite complexity and outgrowth in cortical projection neurons (Pensold et al., 2017).

### Neuronal differentiation

Rac1 and Cdc42, which are downstream of NGF, are necessary for regulating various aspects of neuronal differentiation (Lamoureux et al., 1997; Yasui et al., 2001). As an effector of Rac1 and Cdc42, PAK1 controls the morphology and differentiation of cortical neurons via regulating cytoskeletal reorganization. PAK1 can also interact with NCAM to activate actin polymerization and promote neuronal differentiation (Li et al., 2013). In mice, PAK3 expression is upregulated as GABAergic interneurons differentiate. High PAK3 expression is associated with increased neurite length and decreased neurite branching of cortical GABAergic interneurons in mice (Cobos et al., 2007). PAK3 is also highly expressed in OPCs (oligodendrocyte precursors) and shows decreased levels in mature oligodendrocytes (Maglorius Renkilaraj et al., 2017). Loss of *Pak*3 gene has reduced the density of differentiated oligodendrocytes in the developing white matter, suggesting that the expression of PAK3 in OPCs may be essential for the transformation from a proliferative to a differentiation stage (Maglorius Renkilaraj et al., 2017). Although the mechanism by which PAK3 promotes OPC differentiation remains unknown, one hypothesis is that PAK3 may control OPC differentiation by regulating the subcellular localization of LIMK1. This is because oligodendrocyte maturation depends on intracellular protein shuttling, and the accumulation of nuclear LIMK1 inhibits OPC differentiation (Gottle et al., 2015). In addition, *Pak4*-null embryos have shown defects in the differentiation of both spinal cord motor neurons and ventral interneurons. In particular, the lack of neuronal differentiation has been observed throughout the neural tube of *Pak4*-null embryos (Qu et al., 2003).

### Axonal development

PAKs can also regulate axonal function and process, such as axonal transport, growth, and guidance. In *Drosophila*, PAK1-regulated actin cytoskeleton is essential for the axon guidance of photoreceptor cells (Hing et al., 1999). In mice, NCAM can interact with PAK1 and promote axonal growth in hippocampal neurons (Li et al., 2013). Other adhesion molecules, such as DSCAM, also interact and activate PAK1 to regulate cell migration and neuronal differentiation and to induce axonal guidance (Rashid et al., 2001). PAK4 also plays an important role in axonal outgrowth and neuronal development by regulating actin cytoskeletal reorganization in neural progenitor cells (Jaffer and Chernoff, 2002; Qu et al., 2003). *Pak4*-deficient mouse embryos displayed impaired axonal outgrowth and neurons, including interneurons and spinal cord motor neurons, which failed to migrate to their proper locations in these mice (Qu et al., 2003).

The GEF domain of UNC-73, a Trio-like guanine nucleotide exchange factor, can specifically interact with Rac and activate UNC-73-Rac-PAK1 signaling, which has been found to contribute to the induction of motor commissural axons in *C*. *elegans* (Lucanic et al., 2006). In mice, TRIO, an UNC-73 mammalian homolog, can bind to DISC1, which results in the activation of TRIO-Rac1-PAK1 signaling that regulates axonal connectivity and guidance in the developing brain (Chen et al., 2011). Rac-PAK signaling also regulates the interaction between Schwann cells and axons in the peripheral nerves (Nakai et al., 2006). Before differentiation, cytoskeleton remodeling in Schwann cells is required for their expansion and migration towards the direction of axons (Nakai et al., 2006). Inhibition of Rac-PAK can restore neuronal interactions and promote aligned processes with neurites in Schwann cells (Nakai et al., 2006). Moreover, loss of MOCA (Modifier of Cell Adhesion), a guanine nucleotide exchange factor for Rac1 and a presenilin binding protein, results in altered PAK signaling, abnormal aggregation of neurofilament proteins, and disorganization of the axonal cytoskeleton, as well as axonal degeneration (Chen et al., 2005). In contrast, activation of MOCA-PAK-LIMK signaling can increase cofilin phosphorylation and have a regulatory effect on actin dynamics to prevent axonal degeneration (Chen et al., 2009). Axonal dysfunction, such as impaired axonal transport in the dystrophic axons and abnormal axonal growth and synaptogenesis, caused by abnormal gene expression or mutations in the Rac-PAK-related signaling pathway, have been observed in patients with schizophrenia, neurodegenerative diseases, and psychiatric disorders (Aston et al., 2005).

### Other functions of PAKs in nervous systems

Neuronal polarity, such as axo-dendritic polarity, which allows the undifferentiated neurites to form the typical neuronal shape with short dendrites and a long axon, is critical to proper neuronal connectivity (Jan and Jan, 2003). FOXO transcription factors are widely activated in the developing mammalian brain and promote a switch from a nonpolarized state to a polarized morphology in neurons (Brunet et al., 1999; Hoekman et al., 2006). PAK1 is a direct target for FOXO transcription factors to control both actin and microtubule dynamics in neurons. In vivo knockdown of FOXO in the developing rat cerebellar cortex induced a robustly downregulation of PAK1 and impaired neuronal polarity in primary granule neurons (de la Torre-Ubieta et al., 2010). Moreover, PAK1 functions downstream of the Par polarity complex Par3/Par6/aPKC, which is also regulated by FOXO proteins (de la Torre-Ubieta et al., 2010). The FOXO-PAK1 pathway represents an important transcriptional mechanism that establishes neuronal polarity (de la Torre-Ubieta et al., 2010).

Precise positioning of dendritic branches is essential for establishing neuronal circuitry during development. Previous studies have shown that the Dscam1 (Down syndrome cell-adhesion molecule), the receptor of anterior corner cells in the motor neurons, can recruit Dock and PAK1 to the plasma membrane and define the precise positioning of dendritogenesis in *Drosophila* (Kamiyama et al., 2015). Similarly, in mammals, PAK1 interacts with the SH2-SH3 domain of the NCK protein, an ortholog of *Drosophila* Dock (Kamiyama et al., 2015). *Pak1* mutation reduces the number of anterior corner cell dendritic branches and causes the misplacement of remaining branches (Kamiyama et al., 2015).

PAKs can regulate nerve conduction in non-neuronal cells. One of the few studies indicates that PAKs regulate the formation of the myelin sheath that wraps around axons in both the CNS (central nervous systems) (Maglorius Renkilaraj et al., 2017) and PNS (peripheral nervous systems) (Hu et al., 2016), the deficits of which can impair the nerve conduction of electrical impulses and have been reported in many neurological diseases. Oligodendrocytes are the myelin-forming cells of the CNS. One study showed that PAK3 was highly expressed at the OPC stage, and its expression decreased in differentiated and mature oligodendrocytes. *Pak3* knockout mice displayed delayed OPC differentiation and consequently myelination defects in the corpus callosum (Maglorius Renkilaraj et al., 2017), suggesting that PAK3 is a new regulator of OPC differentiation. Schwann cells form the myelin in the PNS. Establishment of Schwann cell polarity includes the regulation of actin cytoskeleton. Schwannomas containing Schwann cells that show high Rac activity and disorganized cytoskeleton structure fail to interact with axons (Nakai et al., 2006). Moreover, increased F-actin levels correlated with enhanced PAK1 activity were found in a mouse model with disruption of myelin junctions in Schwann cells (Hu et al., 2016). The abnormal F-actin levels, myelin junction disruption, and nerve conduction failure in the mice can be completely rescued by pharmacological inhibition of PAK1 (Hu et al., 2016), suggesting a potential therapeutic approach for demyelinating diseases.

## Neurological diseases associated with pak dysfunction

Due to their crucial function in neural cytoskeleton regulation, PAKs play an indispensable role in brain development and behavior, and are involved in diverse neurological diseases, including both neurodevelopmental and neurodegenerative diseases. The identified mutations of PAKs in diverse neurological diseases have been summarized in Table 3.

**Table 3:** Rodent models of *Pak* members

Genotype	Features	Phenotypes	Impaired Signals	References
*Pak1*^−/−^	A part of the ATG exon and adjacent upstream intronic sequence were replaced by a PGK-NRG cassette	Deficit in E/I balance, deficit in LTP	↓synaptic COX-2 levels, ↑tonic AEA signaling, ↓p-CFL	Xia et al., 2016; Asrar et al., 2009
*Pak2*^−/−^	*Pak2* knockout	Lethal at E8.5	–	C.H., Z. M. Jaffer and J.C., unpublished
*Pak2*^*+*/−^	A poly-A and a PGK promoter were inserted before the exon 2 of *Pak2* to terminate the transcription	Autism-related behaviors	↓pLIMK1/CFL	Wang et al., 2018
*Pak3*^−/−^	A part of the coding and adjacent downstream intronic sequence were replaced by a PGK-NRG cassette	Defects in LM, myelin defects in CC	–	Meng et al., 2005; Maglorius Renkilaraj et al., 2017
*Pak1*^−/−^;*Pak3*^−/−^	DKO	LM defects; hyperactivity	↓p-CFL	Huang et al., 2011
*Pak4*^−/−^	The exon 1 was replaced with a PGK-NPG cassette flanked with 5’ and 3’ homology regions.	Lethal at E10.5; heart and neural tube defects	–	Qu et al., 2003
*Pak5*^−/−^;*Pak6*^−/−^	DKO	Impaired learning and locomotion	–	Nekrasova et al., 2008

Note: PGK-NRG, PGK-neomycin resistant gene; E/I, excitation and inhibition; LTP, long-term potentiation; CC, corpus callosum; LM, learning & memory; E, embryonic day; p-CFL, phospho-cofilin; DKO, double knockout; –, not determined

### Autism spectrum disorders (ASDs)

ASDs are a group of complex neurodevelopmental disorders characterized by abnormal social interaction and communication and restricted repetitive behavior (de la Torre-Ubieta et al., 2016). To date, approximately 1000 ASD-related genes have been added to the database (Pereanu et al., 2018), suggesting the high heterogeneity of this disease. Several recent studies have demonstrated the pathogenic mechanisms of PAK dysfunction and the impairment of its regulated-cytoskeletal dynamics in ASD etiology. For example, we found that *PAK2* haploinsufficiency resulted in autism-related behaviors in both mice and humans. PAK2 dysfunction decreased the phosphorylation levels of LIMK1 and cofilin and their regulated actin networks related to ASDs (Wang et al., 2018). Functional annotations of PAK2-regulated genes have shown that actin dysregulation represents a common pathophysiological mechanism in ASD (Wang et al., 2018). Moreover, Deficiency of *Shank3*, a known autism candidate gene that encoded synaptic scaffold, caused a strong in F-actin and NMDAR-mediated synaptic current by interfering with PAK signaling, which resulted in synaptic dysfunction and abnormal social behaviors in mice (Duffney et al., 2013). Notably, PAK1-mediated actin dynamics can partially rescue ASD-related synaptic and behavioral phenotypes induced by *Shank3* mutations in mice (Bozdagi et al., 2010; Peca et al., 2011). Recently, gain-of-function *PAK1* mutations have also been identified in patients with neurodevelopmental phenotypes, including intellectual disability, macrocephaly, speech delay, seizures, and seizures (Harms et al., 2018; Horn et al., 2019). The finding suggests the direct contribution of PAK signaling to the pathology of the neurodevelopmental disease.

### Alzheimer’s disease (AD)

AD is characterized by the accumulation of excessive Aβ (amyloid beta) and tau proteins, which trigger a complex cascade resulting in synaptic loss and neurotransmitter deficiencies (Sivanesan et al., 2013). When Aβ accumulates in the brain, it interacts with PAKs by binding to p75^NTR^ (a nerve growth factor receptor) to promote the activation of PAKs and phosphorylation of cofilin, eventually promoting NMDA excitotoxicity, a trigger of AD (Gu et al., 2014). A substantial loss of total PAKs has been detected in the cortex and hippocampus of patients with advanced AD (Zhao et al., 2006; Ma et al., 2008; Nguyen et al., 2008). Moreover, aberrant translocation of PAK1 from cytoplasm to membrane and reduced postsynaptic PAK3 levels were also found in the brains of AD patients (Ma et al., 2008; Lauterborn et al., 2020), suggesting that PAK dysfunction impaired the stabilization of actin network and resulted in deficiencies in synaptic plasticity and behaviors. In the mouse model, PAK levels varied with age and were associated with the progression of AD (Ma et al., 2008). Loss of PAK expression specifically in the forebrain altered cortical synaptic morphology and prevented memory consolidation in mice (Dai et al., 2014). Considering the molecular pathways involved in PAK signaling are critical to synaptic function, PAKs are considered potential therapeutic targets for AD. Notably, although PAK inactivation decreased drebrin expression and impaired social recognition, it failed to further enhance Aβ/tau pathology in AD mice (Arsenault et al., 2013). Thus, the reduction of PAKs is a consequence of the neuropathology of AD rather than a cause; thus, therapeutic activation of PAKs may only have symptomatic benefits for brain function in AD (Arsenault et al., 2013).

### Mental retardation (MR)

Fragile X syndrome (FXS), the most common genetic form of MR, is caused by repeated CGG amplification of the *FMR1* (fragile X mental retardation 1) gene on the X chromosome (O’Donnell and Warren, 2002; Darnell et al., 2011). Patients with FXS showed mental retardation, language disorder, appearance change, and behavioral disorder (Darnell and Klann, 2013). Similarly, an animal model of FXS exhibited multiple neuronal and behavioral abnormalities (Bhattacharya et al., 2012). Previous studies have consistently found that impaired RAC/PAK signaling and abnormal PAK expression were associated with abnormal dendritic spine architecture, neuronal development, and aberrant behaviors in *Fmr1* mice (Chen et al., 2010; Santini et al., 2017). In *Fmr1* mice, TBS (theta-burst stimulation) failed to activate and induce PAK phosphorylation in the spine, and newly formed actin filaments induced by theta stimulation remained unstable (Chen et al., 2010). Correspondingly, TBS induced-Schaffer-commissural fEPSP (fleld exciatatory postsynaptic potential) slopes were vulnerable to the treatment that disrupts dynamic actin filaments and showed a gradual decay in a short period, suggesting that LTP stabilization was abnormal in the mutants (Chen et al., 2010). Due to the lack of FMRP, CYFIP1 cannot inhibit Rac1 activity in *Fmr1* mice, resulting in activation of the Rac1-PAK pathway and actin dynamic changes (Santini et al., 2017). Unusually elevated Rac1-PAK1 signaling results in a balance breakdown (F-actin/G-actin) of the actin cytoskeleton and an immature spine phenotype in *Fmr1* mice (Pyronneau et al., 2017). Notably, the morphological and behavioral phenotypes of *Fmr1* mice are rescued either by expressing a dominant-negative form of PAK in the forebrain or by pharmacological inhibition of PAK (Hayashi et al., 2004; Hayashi et al., 2007; Dolan et al., 2013), suggesting the therapeutic potential of PAKs.

PAK3-related mental retardation also represents a rare cause of X-linked MR. Several loss of function and splice mutations in the *PAK3* gene have been identified in different families of X-linked mental retardation with certain specific clinical features, such as microcephaly, mild to moderate mental retardation, oral motor dysfunction and aggressive behavior (Allen et al., 1998; Bienvenu et al., 2000; Gedeon et al., 2003; Peippo et al., 2007; Arsenault et al., 2013). Notably, clinical features usually observed in MR, such as autistic features, epilepsy or sleep disorder, have not been frequently observed in PAK3-related disorders (Rejeb et al., 2008), suggesting the distinguishable phenotypes of PAK3-related MR from other X-linked MR syndromes.

### Schizophrenia

Schizophrenia is a severe neuropsychiatric disorder with complex polygenic genetic patterns and affects approximately 1% of the world population (Lewis and Levitt, 2002; Ross et al., 2006). Patients with schizophrenia showed neurologic symptoms (delusions, hallucinations, and lack of motivation), and cognitive symptoms (poor executive functioning, trouble thinking, and working memory deficits) (Glantz and Lewis, 2001; Lewis et al., 2003; Sullivan et al., 2003). Several studies consistently found that the brain of patients with schizophrenia showed a reduction in dendritic spine density (Glantz and Lewis, 2001). Recent exome sequencing studies of *de novo* in schizophrenia trios and disruptive mutations in schizophrenia sporadic cases have revealed that genes related to activity-regulated cytoskeleton complex contributed to the high ranked genesets for schizophrenia (Fromer et al., 2014; Purcell et al., 2014), suggesting that dysfunction of actin skeletal dynamics contributed to the loss of dendritic spines in schizophrenia. In the ACC (anterior cingulate cortex) and DFC of patients with schizophrenia, PAK1 phosphorylation levels were reduced (Rubio et al., 2012). PAK1 phosphorylation inhibits MLCK (myosin light chain kinase), an enzyme that phosphorylates MLC (Sanders et al., 1999). However, the levels of pMLC (phosphorylated MLC), which is crucial for the structural actin cytoskeleton stability, were only increased in the ACC, and its expression in the DFC remained unchanged, suggesting that the PAK1 downstream pathways are distinguishingly affected in the cortical regions of patients with schizophrenia (Rubio et al., 2012). The increase in pMLC levels in the ACC has been linked to the shrinkage of the dendritic spine, suggesting a PAK1-related mechanism that regulates dendritic spine loss in schizophrenia (Rubio et al., 2012). Moreover, the schizophrenia risk gene *DISC1* affects axonal guidance by activating the Rac-PAK signaling pathway (Kamiya et al., 2005). In patients with schizophrenia, abnormal expression of DISC1 can decrease PAK activity and cause defects in axonal guidance and neuronal connectivity (Chen et al., 2011). *GIT1*, a gene that regulates actin filament dynamics associated with schizophrenia, can bind to Rac/Cdc42 and interacts with PAKs. Most schizophrenia cases carrying *GIT1* variants fail to induce PAK3 activation and GAD1, the key enzyme in GABA biosynthesis, in neurons (Kim et al., 2017), suggesting the contribution of PAK3 dysregulation in the synaptic deficits in schizophrenia.

### Parkinson’s disease

Parkinson’s disease is a movement disorder characterized by the degeneration of dopaminergic neurons in the substantia nigra and reduction of dopamine in the striatum (Dauer and Przedborski, 2003). Among group II PAKs, PAK4 activity was significantly reduced in the substantia nigra of patients with and rodent models of Parkinson’s disease (Won et al., 2016). Decline of PAK4 expression and activity in the human midbrain during aging can lead to a pre-parkinsonian state (Won et al., 2016). Overexpression of constitutively active PAK4 can protect dopaminergic neurons and preserve motor function (Won et al., 2016). PAK4 may promote the survival of dopaminergic neurons through the AKT signaling pathway (Kuijl et al., 2007; Tyagi et al., 2014). As an endogenous neuroprotective kinase, PAK4 and the PAK4-CRTC1^S215^-CREB pathway may be useful therapeutic targets for alleviating the symptoms of Parkinson’s disease (Won et al., 2016). Moreover, PAK6 can bind to LRRK2 (leucine-rich repeat kinase 2), which is a cause of Parkinson’s disease, and is required for LRRK2 to regulate neurite outgrowth (Civiero et al., 2015). The aberrant activation of PAK6 in the striata of patients with LRRK2-linked Parkinson’s disease supports the role of PAK6 in the pathogenesis of Parkinson’s disease (Civiero et al., 2015).

### Neurofibromatosis type 1

NF1 (neurofibromatosis type 1) has an autosomal dominant pattern of inheritance (Barton and North, 2004; Noll et al., 2007). One altered copy of the *NF1* gene can cause the disease (Pride et al., 2014). Over half of patients with NF1 have deficits in social behaviors and social information processing (Huijbregts et al., 2010; Huijbregts and de Sonneville, 2011), and around one-third of patients present with a severe clinical diagnosis of ASD (Garg et al., 2013; Walsh et al., 2013). Further analyses of patients with NF1 and social deficits showed that they also had problems with facial emotion recognition, a function linked to the human amygdala (Huijbregts et al., 2010; Garg et al., 2013; Lehtonen et al., 2013). In mice with a loss of a single *NF1* allele, neurofibromin levels were reduced in several brain regions, including the hippocampus, amygdala, and frontal cortex, as observed in patients with NF1 (Costa et al., 2002; Cui et al., 2008; Molosh et al., 2014). Moreover, abnormal GABA and glutamate neurotransmission and MAPK pathways, disruptive LTP, and loss of expression of the synaptic protein disintegrin and Adam22 (Fukata et al., 2006) and Hsp70 (Molosh et al., 2014) were found in the amygdala of mice. Notably, although no obvious alterations in PAK activity or expression were observed, the social behavior deficits and amygdala disruptions in *Nf1*^+/−^ mice can be rescued by pharmacological blocking PAK1 activity in the amygdala or by the additional deletion of the *Pak1* gene (Molosh et al., 2014), suggesting an indirect contribution of PAKs to the disease. Further study should investigate the molecular mechanism underlying the interaction between PAK1 and NF1 to better support the potential therapeutic role of PAKs in NF1.

### 3q29 microdeletion syndrome

3q29 microdeletion syndrome is a rare chromosomal disorder caused by the deletion at the long (q) arm of chromosome 3. Approximately 80%–99% of people with the 3q29 microdeletion have mild to moderate intellectual disability. They also have an increased risk of behavioral or psychiatric disorders, including autism, bipolar disorder, and schizophrenia (Willatt et al., 2005). The most frequently deleted segment of chromosome in patients with 3q29 microdeletion contains about 20 genes (Quintero-Rivera et al., 2010). *De novo* copy-number variations that contain *PAK2* were found in children with 3q29 microdeletion syndrome (Willatt et al., 2005). Notably, our study also found another *de novo* copy-number deletion containing the *PAK2* gene in a patient with ASD (Wang et al., 2018), suggesting the contribution of *PAK2* gene deletion in 3q29 microdeletion syndrome.

## Conclusions and perspectives

PAKs are widely present in neuronal and non-neuronal cells in the central and peripheral nerve system. In neuronal cells, each *PAK*/*Pak* shows different expression pattern thus exerts differential roles during brain development. *Pak*s are also highly expressed in non-neuronal cells, such as oligodendrocytes, astrocytes, and peripheral glial cells. In particular, PAKs in oligodendrocytes have found to regulate myelin sheath formation to contribute to nerve conduction. Thus, whether the PAK-regulated cytoskeleton in non-neuronal cells (such as oligodendrocyte and astrocytes) affects neuronal function and how it contributes to neurodevelopment and brain function requires further investigation.

PAKs regulate synaptic plasticity by regulating both spine morphology and synaptic transmission/trafficking. PAKs can also regulate diverse neural functions and processes and are involved in broad neural activities in the brain. PAKs participate in various signaling pathways related to cytoskeletal remodeling and thus are involved in the molecular pathology of neurological disorders at different neurodevelopmental stages. PAKs thus have been found to play both direct (such as ASD, MR and AD) and indirect roles (NF1) in the pathology of neurological diseases. Although some of the molecular mechanisms remain unknown, PAKs, possessing specific small-molecule inhibitors in cancer treatment, also represent potential therapeutic targets of neurological diseases involving PAK dysfunction, which necessitates further investigation. Moreover, considering the differential roles of PAKs in neural function, small molecules specific for each PAK rather than a group of PAKs may provide more precise and accurate improvement for PAK-related neurological diseases.
